# Suicidal Women

**Published:** 1859-02

**Authors:** 


### SUICIDAL WOMEN.

Unwise above many is the man who considers every hour lost which is not spent in reading, writing, or in study; and not more rational is she who thinks every moment of her time lost which does not find her sewing.

We once heard a great man advise that a book of some kind be carried in the pocket to be used in case of any unoccupied moment. Such was his practice. He died early and fatuitous!

There are women who, after a hard day's work, will sit and sew by candle or gas light until their eyes are almost blinded, or until certain pains about the shoulders come on which are almost insupportable, and are only driven to bed by a physical incapacity to work any longer. The sleep of the overworked, like that of those who do not work at all, is unsatisfying and unrefreshing, and both alike wake up in weariness, sadness and languor, with an inevitable result, both dying prematurely.

Let no one work in pain or weariness. When a man is tired he ought to lie down until he is most fully rested, when with renovated strength the work will be better done, done the sooner, done with a self-sustaining alacrity.

The time taken from seven or eight hour's sleep out of each twenty-four is time not gained, but time more than lost; we can cheat ourselves, we cannot cheat nature. A certain amount of food is necessary to a healthful body, and if less than that amount be furnished, decay commences the very hour. It is the same with sleep, and any one who persists in allowing himself less than nature requires, will only hasten his arrival at the madhouse or the grave.

